# Crystal Plasticity Simulation of Cyclic Behaviors of AZ31B Magnesium Alloys via a Modified Dislocation–Twinning–Detwinning Model

**DOI:** 10.3390/ma18010025

**Published:** 2024-12-25

**Authors:** Yingjun Sun, Ke Yue, Chongzhi Lin, Zhe Liu, Xiaochuan Sun, Guoliang Wang, Huamiao Wang

**Affiliations:** 1 Chengdu Institute of Advanced Metallic Material Technology and Industry Co., Ltd., Chengdu 610300, China; sunyingjun0036@163.com (Y.S.); yjyyueke@pzhsteel.com.cn (K.Y.); linchongzhi@163.com (C.L.); 2 Guangdong Provincial Key Laboratory of Material Joining and Advanced Manufacturing, China-Ukraine Institute of Welding, Guangdong Academy of Sciences, Guangzhou 510650, China; 3 State Key Laboratory of Metal Matrix Composites, School of Materials Science and Engineering, Shanghai Jiao Tong University, Shanghai 200240, China; garaireen@sjtu.edu.cn; 4 Key Laboratory of Solid State Physics and Devices Autonomous Region, School of Physics Science and Technology, Xinjiang University, Urumq 830046, China; wohennagu0@163.com; 5 Shanghai Key Laboratory of Mechanics in Energy Engineering, Shanghai Frontier Science Center of Mechanoinformatics, Shanghai Institute of Applied Mathematics and Mechanics, School of Mechanics and Engineering Science, Shanghai University, Shanghai 200072, China

**Keywords:** magnesium, cycle deformation, modeling, crystal plasticity, detwinning

## Abstract

In this study, a probabilistic model within the dislotwin constitutive framework of DAMASK (the Düsseldorf Advanced Material Simulation Kit) was established to describe the cyclic loading behaviors of AZ31B magnesium alloys. Considering the detwinning procedure within the twinned region, this newly developed dislocation–twinning–detwinning model was employed to accurately simulate stress–strain behaviors of AZ31B magnesium alloys throughout tension–compression–tension (T-C-T) cycle loading. The investigations revealed that the reduction in yield stress during the reverse loading process was attributed to the active operation of twinning and detwinning modes. Furthermore, the evolution of the twin volume fraction during cycle loading scenarios was quantitatively determined. According to these results, the relative activities of plastic deformation modes during T-C-T loading were further analyzed.

## 1. Introduction

Magnesium alloys have received substantial attention in both scientific and engineering fields as the lightest structural material. However, they exhibit poor room-temperature formability, associated with a lack of insufficient slip modes. Considerable experimental and modeling efforts were undertaken to elucidate the correlation between mechanical behaviors and deformation mechanisms [1,2,3]. It was revealed that the mechanical behavior is severely prompted through the twinning-relevant mechanisms, such as the exclusive *c*-axis strain accommodation by twinning deformation. Therefore, several researchers have extensively investigated the influence of twinning on the deformation responses of magnesium alloys [4,5,6]. These studies comprehensively demonstrated that deformation twinning could be the primary deformation mechanism at low temperatures (<200 °C), due to its lower critical resolved shear stress (CRSS) compared to the slip [2,7].

It is commonly realized that twinning is initially induced by the shearing of a portion of atomic planes [8]. As a consequence, both twin and parent lattices are in mirror symmetry with respect to the twinning plane [8,9,10,11,12,13]. Due to its characteristic polar nature, the evolution of a deformation twin is generally accepted to involve three fundamental stages: nucleation, propagation, and thickening [14,15,16]. Remarkably, an in situ neutron diffraction experimental examination evidences that most twins would shrink and eventually disappear under a reverse loading, i.e., the so-called detwinning phenomenon [17,18]. Further studies on cyclic loading demonstrated that the plastic deformation was predominantly determined by the twinning-detwinning phenomenon [19,20,21]. This was quite reasonable because the twinned grains favored detwinning under the subsequent reverse loading. Therefore, detwinning can be considered as re-twinning in the initially twinned area (first twin) with a specific twin variant.

Several crystal plasticity models incorporating both twinning and slip have been presented to illustrate the mechanical behaviors of various metals. These models can accurately simulate both stress–strain responses and grain orientation changes associated with deformation twinning [22,23,24,25,26]. For instance, Kalidindi et al. integrated deformation twinning into the classical crystal plasticity model and further employed it on simulating both the texture evolution and strain hardening of face-centered cubic crystalline materials [27]. In this model, the evolution of twin volume fraction (TVF) was defined by a simple power law, which was analogous to that of a slip [27,28]. Wong et al. also developed a physically based crystal plasticity model including phase transformation and twinning for high Mn steel, which was implemented within the crystal plasticity computational framework DAMASK (Düsseldorf Advanced Material Simulation Kit) [29,30]. In its twinning illustration, the twin was considered as multiple thin layers, with their sizes described by the parameter of the mean free path (MFP). In addition, these crystal plasticity models can be applied in the investigations on hexagonal closed packed (HCP) metals [31]. For instance, Yaghoobi et al. examined the twinning response of the WE43 magnesium alloy via a combination of SEM-DIC observation and crystal plasticity finite element (CPFE) simulation [32]. The simulated twin distribution in this multiscale rate-independent model exhibited excellent concordance with the experimental data. However, these twinning models merely consider the nucleation and subsequent growth process, with limited prediction capabilities for detwinning-relevant processes [33,34,35,36,37,38].

Based on the phenomenological description of stress–strain curves, several researchers investigated the twinning–detwinning behaviors via various macroscopic plastic models [24,25,39,40]. These models cannot provide intuitive microstructure information, such as TVF, texture evolution, and activation criteria of various plastic deformation modes. Fortunately, the crystal plasticity method is highly effective in describing the microstructure evolution. For instance, the behaviors of twinning and detwinning were simulated by an elastic viscoplastic self-consistent (EVPSC) model [41,42,43]. In this twinning and detwinning (TDT) model, twins are treated as fresh grains [44,45]. When the stress direction resolved on the twinning system was reverse relative to the twinning direction, detwinning could occur and thus lead to a decrease in the twin volume [46,47]. The relationship between the shear rate and stress of twinning (or detwinning) was analogous to that of the slip. The TVF could be calculated from the cumulative shear strain produced by twinning. Upon reaching a threshold value of TVF, this grain orientation was rotated to a new orientation associated with this twinning system. When the cumulative shear strain produced by detwinning reached a threshold value, this twinned grain would reorient back to its initial parent orientation. However, these models mainly employ phenomenological approaches to describe the detwinning behaviors, which fail to capture its microstructural characteristics. Limited attention was given to the cycle deformation simulation of magnesium alloys via the detwinning-relevant physical model. Therefore, more sophisticated simulation approaches are required to enhance the model’s predictive abilities via incorporating the detwinning procedure.

In this research, a physically based detwinning model was initially established and further implemented in DAMASK. Based on this new model, the cycle deformation behaviors under tension and compression were comprehensively simulated. Both TVF evolution and relative activities of several deformation mechanisms (including slip, twinning, and detwinning) during cycle deformation have been analyzed.

## 2. Experimental

### 2.1. Materials

**Figure 1:**
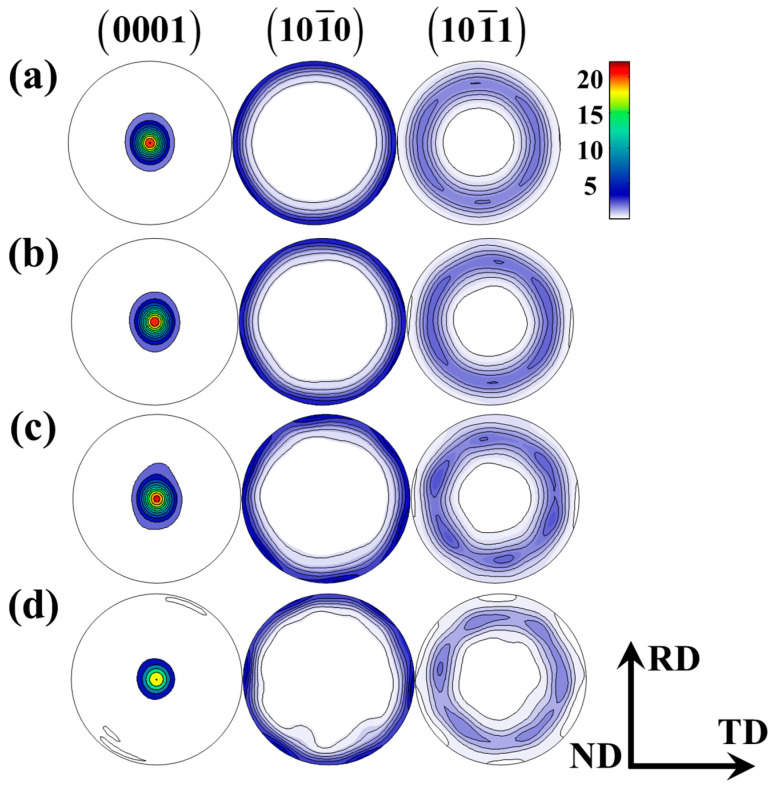
Pole figures displaying the initial orientations of AZ31B magnesium alloy plate: (**a**) experimental data from Lou et al. [2]; (**b**) randomly selected dataset with 1728 grains; (**c**) randomly selected dataset with 512 grains; and (**d**) randomly selected dataset with 125 grains.

The original material was a commercially rolled AZ31B magnesium alloy plate. As illustrated in Figure 1a, the original texture of this magnesium alloy was determined through an XRD measurement. The measured pole figure clearly exhibited a representative basal texture with its basal planes nearly vertical to the normal direction (ND) and the prismatic poles randomly rotated around the ND. The orientation data were used to generate three groups of discrete Euler angles for the input texture in the CPFE simulation. Specifically, 1782, 512, and 125 orientations were randomly selected and further assigned to the equivalent RVEs. The corresponding pole figures are shown in Figure 1b–d. It is clearly detected that the pole figures containing 1728 and 512 grains substantially represent the measured texture given in Figure 1a, while the pole figure containing 125 grains demonstrates a noticeable deviation. However, such a texture deviation cannot significantly affect the stress–strain response, which will be thoroughly discussed later. The sheet coordinates were defined as follows: RD//*y*-axis, TD//*x*-axis, and ND//*z*-axis. Here, TD and RD represent the transverse direction and rolling direction, respectively.

### 2.2. Mechanical Testing

Uniaxial tensile, uniaxial compression, and cyclic deformation tests were conducted on the AZ31B magnesium alloy plate. The details of these loading processes were described in Lou’s previous work [2].

## 3. The Dislocation–Twinning–Detwinning (DTD) Model

### 3.1. Kinematics

The CPFE model is established within a finite strain framework. The entire deformation gradient tensor F is multiplicatively decomposed as follows:

(1) $$F=F_{e}F_{p}$$

where $F_{e}$ denotes the elastic component correlated with elastic distortion and rotation and $F_{p}$ represents the plastic part responsible for crystallographic slip, twinning, and detwinning. Note that the orientation of the crystal lattice cannot be altered by the crystallographic slip, even if the corresponding $F_{p}$ generally includes a rotational component ($R_{p}$). Also, the plastic deformation gradient rate ${\dot{F}}_{p}$ can be described as follows:

(2) $${\dot{F}}_{p}=L_{p}F_{p}$$

Specifically, the plastic velocity gradient $L_{p}$ is influenced by the slip rates ${\dot{\gamma }}^{\alpha }$ of all active slip systems α and the twinning rates ${\dot{\gamma }}^{\beta }$ of all active twinning (or detwinning) systems β. The comprehensive definition of $L_{p}$ is determined in the subsequent equation:

(3) $$\begin{array}{l} L_{p}=\left(1-\sum_{\beta =1}^{N_{T-D}}f^{\beta }\right)\sum_{\alpha =1}^{N_{S}}{\dot{\gamma }}^{\alpha }m_{S}^{\alpha }⊗n_{S}^{\alpha }+\sum_{\beta =1}^{N_{T-D}}\gamma_{T-D}{\dot{f}}^{\beta }m_{T-D}^{\beta }n_{T-D}^{\beta }+\\ \sum_{\beta =1}^{N_{T}}f^{\beta }\sum_{\alpha =1}^{N_{S}}{\dot{\gamma }}^{\beta ,\alpha }Q^{\beta }m_{S}^{\alpha }⊗n_{S}^{\alpha }Q^{\beta ,T} \end{array}$$

In the above equation, three terms represent individual contributions of the slip, twinning, and slip within the twin. $f^{\beta }$ is the volume fraction of twins within the twinning (or detwinning) system β. The vectors $m_{S}^{\alpha }$ ($m_{T-D}^{\beta }$) and $n_{S}^{\alpha }$ ($n_{T-D}^{\beta }$) represent the slip (twinning or detwinning) direction and the slip (twinning or detwinning) plane normal of the slip system α (twinning or detwinning system β), respectively. $N_{T}$ and $N_{S}$ correspond to the overall amount of activated twinning and slip systems, respectively. $N_{T-D}$ denotes the total number of activated twinning (or detwinning) systems β, which is equal to the value of $N_{T}$. $\gamma_{T-D}$ represents the specific twinning shear strain of extension twinning in magnesium alloys, i.e., 0.129. ${\dot{f}}^{\beta }$ is the evolution rate of TVF in the twinning system β. ${\dot{\gamma }}^{\beta ,\alpha }$ is the slip rate of slip systems α in the twinning system β. $Q^{\beta }$ denotes the rotation matrix of the twinning system β. This process has been reported in previous works, such as in high-Mn steels and HCP metals [48,49].

### 3.2. Constitutive Relationships of DTD Model

According to the CPFE framework established by Huang et al. [50], the shear rate of a single slip system is correlated with the Schmid law using a power law. Furthermore, the dislocation model described by the Orowan equation is preferred due to its enhanced accuracy and extensive usage, as it effectively captures the microscopic features of plastic deformation [30,51]. It is generally identified that the plastic behavior is closely associated with the edge dislocation density $\rho_{e}$. In this DTD model, the constitutive relationship between shear rates of slip and stress is defined as follows:

(4) $${\dot{\gamma }}^{\alpha }=\rho_{e}b_{S}^{\alpha }\nu_{0}\exp\left[-\frac{Q_{S}}{k_{B}T}{\left(1-{\left(\frac{\left|\tau_{eff}^{\alpha }\right|}{\tau_{P}^{\alpha }}\right)}^{p}\right)}^{q}\right]$$

where $b_{S}^{\alpha }$ represents the Burgers vector length of the slip system α, $\nu_{0}$ is the velocity of dislocation motion, $Q_{S}$ denotes the activation energy for dislocation slip, $k_{B}$ is the Boltzman constant, T is the absolute temperature, $\tau_{eff}^{\alpha }$ is the effective resolved shear stress on the slip system α, $\tau_{P}^{\alpha }$ represents the Peierls stress, and p and q are fitting parameters.

The evolution rate of edge dislocation density ${\dot{\rho }}_{e}^{\alpha }$ is further described as follows:

(5) $${\dot{\rho }}_{e}^{\alpha }=\frac{\left|{\dot{\gamma }}^{\alpha }\right|}{b_{S}^{\alpha }\Lambda_{S}^{\alpha }}-\frac{2{\hat{d}}_{e}^{\alpha }}{b_{S}^{\alpha }}\rho_{e}^{\alpha }\left|{\dot{\gamma }}^{\alpha }\right|-\frac{2{\overset{\vee }{d}}_{e}^{\alpha }}{b_{S}^{\alpha }}\rho_{e}^{\alpha }\left|{\dot{\gamma }}^{\alpha }\right|$$

Here, $\Lambda_{S}^{\alpha }$ represents the MFP of slip system α. The MFP of the slip refers to the average distance a dislocation can move freely before encountering an obstacle, such as another dislocation or a twin boundary; ${\hat{d}}_{e}^{\alpha }$ and ${\overset{\vee }{d}}_{e}^{\alpha }$ are two parameters controlling the dipole formation and dislocation annihilation, respectively. It is generally accepted that multiple structures, including grain boundaries, dislocations, and twin boundaries, are observed to effectively inhibit dislocation motion. Therefore, the MFP of a single slip system can be described by the subsequent three factors:

(6) $$\frac{1}{\Lambda_{S}^{\alpha }}=\frac{1}{d_{grain}}+\frac{1}{\lambda_{S}^{\alpha }}+\frac{1}{\lambda_{S-T}^{\alpha }}$$

(7) $$\frac{1}{\lambda_{S}^{\alpha }}=\frac{1}{i_{S}}{\left(\sum_{\alpha^{\prime }=1}^{N_{s}}\zeta_{\alpha \alpha^{\prime }}\rho_{e}^{\alpha^{\prime }}\right)}^{1/2}$$

(8) $$\frac{1}{\lambda_{S-T}^{\alpha }}=\sum_{\beta =1}^{N_{T}}\zeta_{\alpha \beta }f^{\beta }\frac{1}{t_{T}\left(1-f_{T,total}\right)}$$

where $\lambda_{S}^{\alpha }$ denotes the moving distance upon encountering another dislocation, $d_{grain}$ represents the grain size, $\lambda_{S-T}^{\alpha }$ is the moving distance upon encountering a twin, $i_{S}$ is a fitting parameter, $f_{T,total}$ represents the whole TVF, and $t_{T}$ denotes the average twin thickness. $\zeta_{\alpha \alpha^{\prime }}$ denotes the interaction matrix between two individual slip systems *α* and *αꞌ*. $\zeta_{\alpha \beta }$ represents the interaction matrix between slip system *α* and twinning system *β*.

In contrast to the slip deformation, deformation twinning causes a rotation of the initial matrix towards a new crystal orientation. The rotation matrix Q expresses the transition of a lattice vector from its initial position to the final position within the twin:

(9) $$Q^{\beta }=n_{T}^{\beta }⊗n_{T}^{\beta }-\delta_{ij}$$

For the twinning system β, $n_{T}^{\beta }$ represents the unit vector normal to the twinning plane and $\delta_{ij}$ denotes Kronecker’s symbol.

As described before, nucleation, propagation, and thickening are three fundamental stages for the twin formation. The nucleation rate of twinning system β (${\dot{N}}_{nucl}^{\beta }$) is expressed as follows:

(10) $${\dot{N}}_{nucl}^{\beta }={\dot{N}}_{0}P_{nucl}^{\beta }$$

Here, ${\dot{N}}_{0}$ represents the number density of the potential twin nucleus formed per unit time and $P_{nucl}^{\beta }$ is the probability of twin nucleation. Electron backscatter diffraction (EBSD) research has demonstrated a preference for extension twin nucleation at grain boundaries. At the continuum scale, modeling twin nucleation necessitates a probabilistic approach as follows:

(11) $$P_{nucl}^{\beta }=1-\exp\left[-\frac{V_{CS}}{k_{B}T}\left(\tau_{r}-\tau^{\beta }\right)\right]$$

where $\tau^{\beta }$ is the resolved shear stress (RSS) on the twin system β and $V_{CS}$ is the cross-slip activation volume. $\tau_{r}$ is the required stress to bring two partials within the critical distance to establish the twin nucleus.

Following twin nucleation, the twin propagation is driven by the applied stress. The twin propagation could also be considered as a probability event, i.e., $P_{prop,T}^{\beta }$, which is expressed as follows:

(12) $$P_{prop,T}^{\beta }=\exp\left[-{\left(\frac{\tau_{prop,T}}{\tau^{\beta }}\right)}^{r}\right]$$

where $\tau_{prop,T}$ represents the threshold stress required for twin propagation and *r* is a fitting parameter. Then, the evolution rate of TVF ${\dot{f}}^{\beta }$ is as follows:

(13) $${\dot{f}}^{\beta }=\frac{1}{V_{grain}}{\dot{N}}_{nucl}^{\beta }P_{prop,T}^{\beta }V^{\beta }$$

Here, $V_{grain}$ is the grain volume and could be regarded as the average grain volume in the calculation. $V^{\beta }$ denotes the volume of the freshly formed twin layer, which could be calculated by the following:

(14) $$V^{\beta }=\frac{\pi }{4}{\left(\Lambda_{T}^{\beta }\right)}^{2}t_{T}$$

where $\Lambda_{T}^{\beta }$ is the MFP of twin propagation.

(15) $$\frac{1}{\Lambda_{T}^{\beta }}=\frac{1}{i_{T}}\left(\frac{1}{d_{grain}}+\sum_{\beta^{\prime }=1}^{N_{T}}\zeta_{\beta \beta^{\prime }}f^{\beta^{\prime }}\frac{1}{t_{T}\left(1-f_{T,total}\right)}\right)$$

Here, $\zeta_{\beta \beta^{\prime }}$ characterizes the interaction among twinning systems and $i_{T}$ is a fitting parameter. It is reported that both grain boundaries and other twin boundaries are potential obstacles for the twin propagation. Therefore, the MFP of twin propagation is controlled by the grain size and characteristics of pre-existing twins.

**Figure 2:**
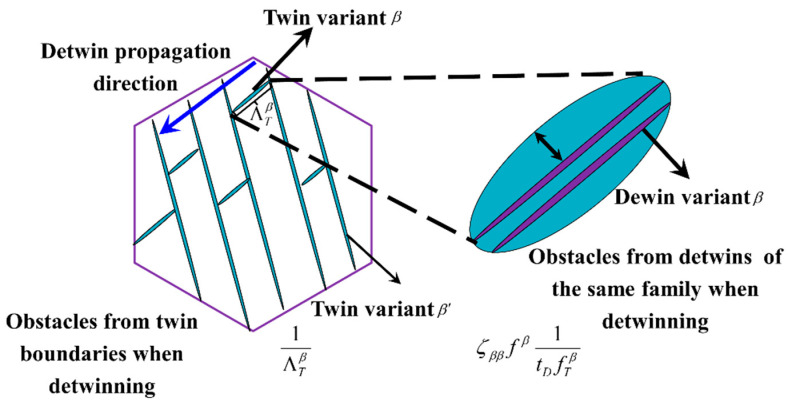
Schematic representation of the MFP of both twin and detwin. The MFP represents the size of a twin or detwin variant. The detwin is the specific twin variant in the twinned area and thus exhibits its own MFP.

The stress threshold essential to initiate detwinning is normally lower than that for twinning. This distinction arises because twinning requires nucleation while detwinning does not. The physical mechanism of detwinning remains a subject of ongoing discussion. One hypothesis posits that detwinning involves the activation of a specific twinning system within the twinned regions, aligning the grain orientation of the re-twinned part closely with the original grain. In the TDT model proposed by Wu and Wang [42,43], the initiation of detwinning is caused by the reverse resolved stress on a twinning system exceeding the threshold value. Hama et al. further indicated that detwinning can be modeled analogously to twinning [52]. Upon the cumulative shear strain from detwinning reaching a threshold value, the twinned grain reorients to its initial orientation. In this paper, detwinning may be activated when the stress direction on a twinning system is reversed (see Figure 2). The subsequent detwin propagation is also controlled by the stress condition. The probability of the detwin propagation $P_{prop,D}^{\beta }$ can be articulated in a manner analogous to twin propagation, as follows:

(16) $$P_{prop,D}^{\beta }=\exp\left[-{\left(\frac{-\tau_{prop,D}}{\tau^{\beta }}\right)}^{r}\right]$$

$\tau_{prop,D}$ is the critical stress for the detwin propagation. Detwinning involves the shrinkage of twinned areas without the requirement for twin nucleation. The evolution rate of TVF due to detwinning can be expressed as follows:

(17) $${\dot{f}}^{\beta }=-\frac{1}{V_{grain}}{\dot{N}}_{nucl}^{\beta }P_{prop,D}^{\beta }\frac{\pi }{4}{\left(\Lambda_{D}^{\beta }\right)}^{2}t_{D}$$

where $\Lambda_{D}^{\beta }$ is the MFP of the detwins. $t_{D}$ is the thickness of the detwin. According to the definition of the MFP of twins, we can define the MFP of detwinning in terms of the following:

(18) $$\frac{1}{\Lambda_{D}^{\beta }}=\frac{1}{i_{D}}\left(\frac{1}{\Lambda_{T}^{\beta }}+\zeta_{\beta \beta }f^{\beta }\frac{1}{t_{D}f_{T}}\right)$$

where $i_{D}$ is a fitting parameter and $\zeta_{\beta \beta }$ is the interaction matrix between the twinning system β and itself. The first term is the obstacles encountered at twin boundaries, while the second term represents the detwinning of the same family. The value of $\Lambda_{D}^{\beta }$ gradually decreases with decreasing TVFs of total twinning systems $f_{T}$. At a calculated TVF of approximately 0.15, the detwinning activity is suppressed and the TVF cannot further reduce. Here, a saturation factor for the twinning volume fraction $f_{sat}$ was introduced to solve this problem.

(19) $$\frac{1}{\Lambda_{D}^{\beta }}=\frac{1}{i_{D}}\left(\frac{1}{\Lambda_{T}^{\beta }}+\zeta_{\beta \beta }f^{\beta }\frac{1}{t_{D}\left(f_{T}^{\beta }+f_{sat}\right)}\right)$$

Following the introduction of $f_{sat}$, the TVF may decrease to 0.05 during the final stage of detwinning. This enhancement in the detwinning model would improve the accuracy in estimating the cyclic deformation.

### 3.3. The Relative Activity of Deformation Mode

The relative activities of various deformation mechanisms are calculated from the accumulated shear rates of these deformation systems. The relative activity for each family, denoted as $R_{i}$, is provided as follows [53]:

(20) $$R_{i}=\frac{\sum_{1}^{N_{grains}}\sum_{\alpha }^{k_{i}}\left|{\dot{\gamma }}^{\alpha }\right|}{\sum_{1}^{N_{grains}}\sum_{\beta }^{N}\left|{\dot{\gamma }}^{\beta }\right|}$$

where $N_{grains}$ denotes the whole numbers of grains, $k_{i}$ represents the amount of slip or twinning of family *i*, and N denotes the summed amount of all slip, twinning, and detwinning systems.

### 3.4. Finite Element Modeling

**Figure 3:**
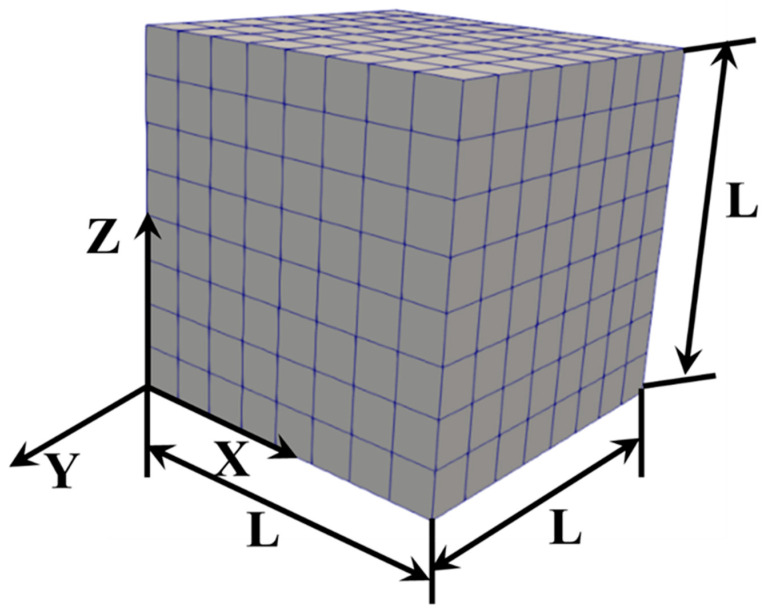
Finite element model consisting of 512 grains in the CPFE simulation. L represents the side length of a regular cube.

The spectral solver implemented in the crystal plasticity framework DAMASK was employed to perform three-dimensional representative volume element (RVE) simulations [30]. To verify the reliability of the RVE implementation, three types of RVEs were considered to represent grid configurations: (I) 12 × 12 × 12, (II) 8 × 8 × 8, and (III) 5 × 5 × 5. Shown in Figure 3 is a typical scenario consisting of 8×8×8 finite elements represented by the 8-node isoparametric brick element. As described in the previous study [54], the surface of this RVE was enforced by the periodic boundary conditions.

Following the experimental procedures of Lou et al. [2], three types of room-temperature deformations along the RD were employed to the RVE with a strain rate of 0.001 $s^{-1}$, i.e., uniaxial tension, uniaxial compression, and tension–compression–tension (T-C-T) tests. Regarding the uniaxial deformation, the corresponding maximum strains under uniaxial tension and compression were 0.15 and −0.09, respectively. The detailed configuration of the cyclic loading process is described as follows: The maximum strain reached 4% in the initial tension step ($\varepsilon_{\max.T}$), followed by a subsequent compression strain of −3.6%, and the simulation was finally terminated when the strain of second tension achieved 2%.

Similar to Lou’s prior research regarding magnesium alloys, the following deformation modes were considered in the present study: basal slip, prismatic slip, pyramidal slip, and extension twinning. The variables in the crystal plasticity model were also determined by the uniaxial tension and compression curves published by Lou et al. [2].

## 4. Results and Discussion

### 4.1. Parameter Identification

The experimental stress–strain curves during the cyclic T-C-T loading were acquired by Lou et al. [2]. Several constitutive parameters of the crystal plasticity model were then determined through fitting the measured stress–strain curves. The parameters relevant to the elastic, slip, and twinning systems are presented in Table 1. Among these calibrated parameters, $i_{S}$ and $\tau_{P}^{\alpha }$ are identified by fitting the stress–strain curve under tension loading, $i_{T}$ and $\tau_{prop,T}$ are derived from the compression loading curve, and $i_{D}$ and $\tau_{prop,D}$ are obtained from the cyclic loading curve. In addition, both $\nu_{0}$ and $Q_{S}$ are referenced from the published paper [29]. Among these parameters, certain parameters are independent of the slip systems, while others are dependent on either slip or twinning systems. Furthermore, the parameters for both slip–twin and slip–slip interactions are evaluated via analyzing relative activation stresses of slip/twinning systems, and the corresponding parameters for slip–twin/slip–slip interactions are shown in Table 2. The slip–twin interaction is linked to the MFP of slip and twinning systems. Due to the larger twin thickness and wider distance between twin lamellae, the interaction coefficients between slip and twinning systems are relatively small. In contrast, the parameters of slip–slip interaction are derived from the relative activities of various slip systems and further determine the correlation between pass stress and dislocation densities. Specifically, the interaction coefficient between basal slip systems is significantly reduced compared to that between pyramidal or prismatic slip systems due to the lower hardening rate of the basal slip.

### 4.2. A Validation of the CPFE Model by Considering Random Initial Texture

**Figure 4:**
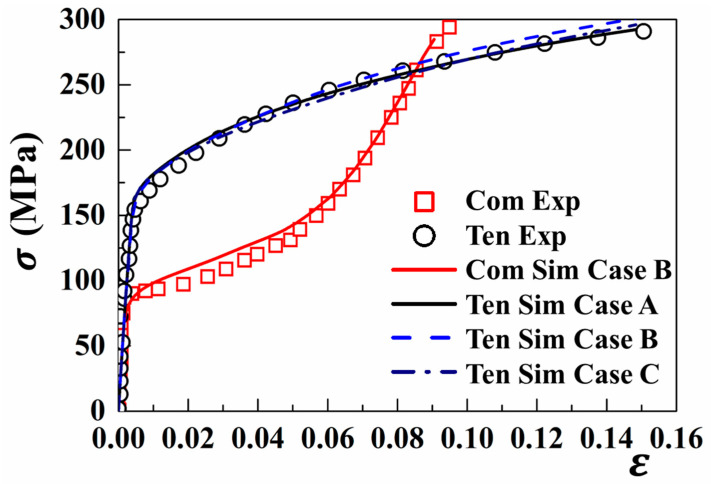
Experimental and simulated stress–strain curves under uniaxial deformation along the RD. The scatter plots are the experimental data from Lou et al. [2], which are represented as “Exp”. The solid lines are the simulation data, which are expressed as “Sim”. “Ten” and “Com” indicate the uniaxial tension and compression along the RD, respectively.

It is generally realized that the mechanical behaviors of polycrystalline materials are associated with the texture features. Regarding the CPFE calculation, a fundamental concern that arises is how to acquire precise simulation results via selecting the appropriate number of grain orientations. To achieve this problem, a random selection of 125, 512, and 1872 grains was randomly extracted from the XRD data of AZ31B magnesium alloys. The corresponding grain sets are identified as Case A, B, and C, with their associated pole figures presented in Figure 1. Subsequently, these grain orientations were randomly assigned to each finite element of three types of RVEs, which were subjected to a uniaxial tension with a strain rate of 0.001 $s^{-1}$ (see Figure 4). The stress–strain curves obtained from 512 and 125 grains under tension are noticeably similar to that of 1872 grains. This further indicates that the selected RVE can effectively capture the mechanical characteristics, and the RVE composed of 512 grains (referred to as Case B) is employed for the cycle deformation.

This developed CPFE model was verified on the macroscale for both tensile and compressive stress–strain curves. The CPFE-predicted stress–strain curves were compared to that received from Lou’s previous work (see Figure 4). As can be seen, the mechanical responses under both tension and compression are accurately captured by the developed CPFE model [52,55]. Specifically, with the increasing tension strain, the hardening rate rapidly increases, followed by a subsequent reduction. Also, the compressive stress–strain curve presents an inflection beyond yielding. Due to the twinning activation inherent to a low resolved stress, a slow increase in the hardening rate is then observed. Following the nearly exhausted twin volume, the hardening rate exhibits a rapid increase caused by the predominant slip deformation mode, which is similar to that during tension. Nevertheless, the estimated results exhibit discrepancies with the experimental data, i.e., the CPFE model slightly overestimates the mechanical responses under tension and compression.

### 4.3. Microscopic Behaviors Under Monotonic Loading

**Figure 5:**
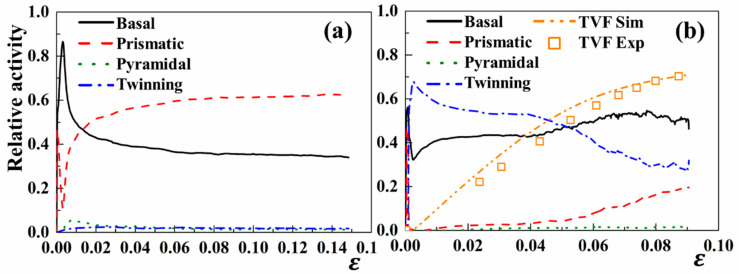
Relative activities of slip and twin modes under (**a**) tension and (**b**) compression along the RD. Both orange dash-dot-dot lines and scatters are the TVF obtained from the CPFE prediction and experimental data, respectively.

The relative activities of various slip and twinning modes during both tension and compression loading are shown in Figure 5. From this figure, both a basal and prismatic slip are observed to be the dominant deformation mechanisms under the RD tension. These two slip systems are activated at low strains of 0.002 and 0.003, respectively. The basal slip is the primary deformation mechanism within the strain range from 0.003 to 0.015, while the prismatic slip develops to be the dominant deformation mechanism beyond a larger strain than 0.015. With the increasing strain, the relative activities of the prismatic and basal slip stabilize around 0.4 and 0.55, respectively. Specifically, the relative activities of extension twinning and the pyramidal slip are much less active during the whole tension deformation.

Compared to the tension scenario, both extension twinning and basal slip were concurrently activated at a compression strain of 0.002. At a larger strain interval between 0.002 and 0.04, the relative activity of the basal slip is approximately 0.4, while that of extension twinning varies from 0.56 to 0.6. Subsequently, the relative activity of extension twinning continues to decrease with the increasing strain. This drop is caused by the increase in TVF and the concurrent reduction in the MFP induced by twin propagation. Furthermore, a reduction in the growth rate of the twin volume is observed and extension twinning essentially reaches saturation at a strain of 0.085. In terms of the prismatic slip, the relative activity starts to slowly increase at a strain of 0.01 and gradually accelerates after the twin saturation. Note that the pyramidal slip is not triggered throughout the entire deformation. This remains in correspondence with the relative activation stresses of various slip/twinning modes described in the literature [44].

The TVF under the compression simulation is also presented in Figure 5b. It can be seen that the TVF increases sharply in the strain interval from 0.00 to 0.05. With the increasing compression strain, the increasing rate of TVF starts to decrease and the TVF approximately saturates at a strain of 0.09. Note that the simulation of extension twinning is comparatively higher than the experimental data sourced from the reference [56].

### 4.4. Prediction of Microscopic Behaviors Under Cyclic Loading

**Figure 6:**
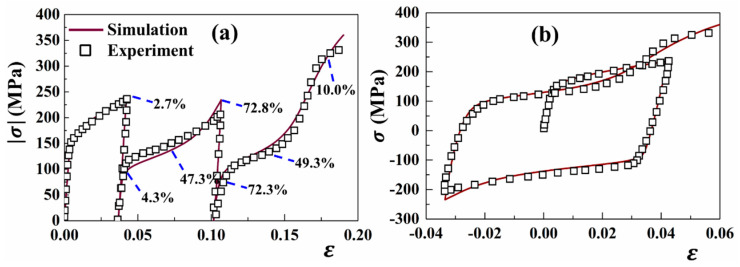
Measured and simulated stress–strain curves under a TCT cycle loading along the RD: (**a**) absolute value; (**b**) original value. The inserted values are the predicted TVFs at various deformation stages.

Both experimental and simulated stress–strain curves under cyclic TCT loading along the RD are shown in Figure 6. It is observed that the estimated curve closely replicates the experimental investigation provided by Lou et al. [2]. During the first loading (or tension), the inactive extension twinning represents a concave-down-shaped stress–strain curve. When the loading direction is reversed (or compression), extension twinning is initiated by a concave-up-shaped flow stress. During the third tension scenario, detwinning is active in the twinned region with a re-emerged concave-up-shaped stress–strain curve. These widely recognized features in the cyclic stress–strain curves are reproduced by the developed CPFE simulation. However, discrepancies between experiments and simulations appear in the plastic stage during the subsequent compression–tension loadings. During the whole T-C-T cycle loading, the yield stress during reverse compression is significantly lower than that during initial tension. This phenomenon is expected to be caused by the lower CRSS required for twinning than other slip modes. Regarding the additional nucleation for twinning compared to twinning, the yield stress under the final tension is further observed to be lower than that under the reverse compression.

The twinning model described here examines the TVF evolution through both propagation and detwinning processes, thereby enabling an analysis of TVF during the cyclic loading (see the inset number in Figure 6a). The accumulated TVF sums over all the twin variants and grains are assumed to be the total TVF. During the initial tension, the TVF increases slightly and reaches 2.7% at the final strain of 0.0417. Following the operation of extension twinning, the TVF rapidly increases to 47.3% (ε = 0.0) and further to 72.8% (ε = −0.033) during the second compression. Afterwards, the operation of detwinning triggers a reduction in the TVF (49.3%) and is finally stabilized at approximately 10.0%. This TVF evolution corresponds closely to the EVPSC simulation observations described by Wang et al. [41]. It is recognized that the TVF would not be eliminated due to the residual twin in the CPFE model. The higher residual TVF leads to an enhanced interaction between residual twins and dislocations, which contributes to a higher yield strength after detwinning.

**Figure 7:**
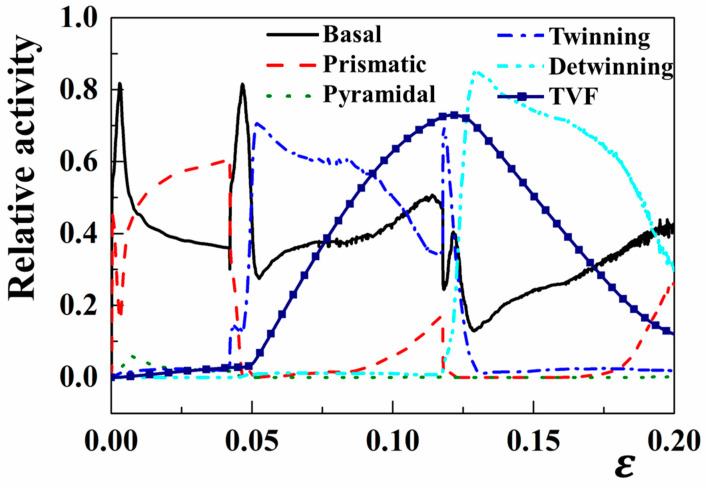
Relative activities of various deformation modes as a function of absolute true strain under a T-C-T cycle loading along the RD.

The activation of slip/twinning modes and their influence on the micromechanical behaviors during cyclic loading were evaluated by investigating the relative activities of various deformation mechanisms. The relative activities associated with the cycle deformation simulation, as an illustration of the plastic strain, are illustrated in Figure 7. The deformation responses under the TCT cyclic loading demonstrate the following features:

(a) During the first loading (tension), the basal slip is initially activated. With the increasing tension strain, the prismatic slip is activated under a large strain of 0.0025 and the relative activity of the basal slip gradually decreased. When the strain exceeded 0.01, the relative activities of the prismatic and basal slip range from 0.46 to 0.6 and 0.36 to 0.46, respectively. Especially, both the pyramidal slip and extension twinning exhibit relatively lower activities, i.e., approximately 0.05.
(b) Under the preliminary stage of the reverse compression, the basal slip is originally activated again and further quickly inhibited with the dominant activity of extension twinning. Simultaneously, the prismatic slip rapidly decreases to a low level. During the latter stage of second loading, the prismatic slip inevitably is activated while the operation of extension twinning gradually diminishes. This phenomenon is a consequence of the grain reorientation induced by extension twinning.
(c) Upon returning to the third tension loading, both basal slip and twinning systems exhibit certain relative activities and then rapidly decrease. The activity of the detwinning mode increases quickly and dominates the deformation up to a strain of 0.05. The relative activity of the basal slip ranges from 0.18 to 0.42 in the reverse tension strain path. The prismatic slip system is activated at a reverse tension strain of 0.02. The enhanced activity of prismatic systems leads to an elevated yield stress during the subsequent stage of the reverse tension loading path.

## 5. Conclusions

In this study, a DTD model was developed to simulate the mechanical behaviors of AZ31B magnesium alloys under a cyclic loading along RD. Furthermore, both the TVF evolution and relative activities of plastic deformation mechanisms were comprehensively examined. The core conclusions are drawn as follows.

(1) The developed CPFE model effectively captures the stress–strain responses under monotonic loadings, i.e., the slip-dominated tension and twin-mediated compression. Three types of RVE selections consistently provide accurate predictions for the CPFE prediction, and an appropriate RVE selection with 512 grains is further utilized to achieve a balance between effectiveness and time consumption. Following the comparison with the reported experimental data, the TVFs calculated by the developed CPFE model exhibit excellent agreement under monotonic loadings.
(2) A probabilistic detwinning growth model effectively captures the activation of both twinning and detwinning during the cyclic deformation. The corresponding evolution of TVF is also reproduced by the CPFE simulation with the implemented detwinning model.
(3) The relative activities of various slip and twinning mechanisms under the cyclic deformation are accurately captured. The prismatic slip is revealed to be the primary deformation mode, and the basal slip assumes a secondary role during the initial tension. Subsequently, twinning and detwinning emerge as primary deformation modes in the second compression and final reverse tension stages, respectively. Simultaneously, the basal slip appears as a secondary incidence during the whole cyclic deformation.

## Figures and Tables

**Table 1:** Input parameters for the constitutive models.

Slip Systems	$b_{S}^{\alpha }$ (m)	$v_{0}$ (m/s)	$Q_{S}$ (J)	$i_{S}$	$\tau_{P}^{\alpha }$ (Pa)	*p*	*q*
Basal	3.2 × 10^−10^	1 × 10^−4^	3.5 × 10^−19^	300	0.8 × 10^7^	1.15	1.0
Prismatic	3.2 × 10^−10^	1 × 10^−4^	3.5 × 10^−19^	15	7.5 × 10^7^	1.15	1.0
Pyramidal	6.11 × 10^−10^	1 × 10^−4^	3.5 × 10^−19^	15	7.5 × 10^7^	1.15	1.0
	$d_{\mathrm{grain}}$ (m)	$\Gamma_{\mathrm{sf}}$ (mJ/J)					
	20 × 10^−6^	0.025					
Twin systems	$i_{T}$	$t_{T}$ (m)	${\dot{N}}_{0}$	r	$\tau_{\mathrm{prop},T}$	$V_{\mathrm{CS}}$	$i_{D}$
	4.0	5 × 10^−8^	4 × 10^3^	3.0	1.2 × 10^7^	1.27 × 10^−29^	1.0
	$t_{D}$ (m)	$\tau_{\mathrm{prop},D}$ (Pa)	$f_{\mathrm{sat}}$				
	3 × 10^−8^	10^7^	0.2				
Elastic modulus	$C_{11}$ (pa)	$C_{33}$ (pa)	$C_{44}$ (pa)	$C_{12}$ (pa)	$C_{13}$ (pa)		
	59.3 × 10^9^	61.5 × 10^9^	16.4 × 10^9^	25.7 × 10^9^	21.4 × 10^9^		

**Table 2:** Interaction parameters between slip and twinning modes.

$\xi_{\alpha \alpha^{\prime }}$	Basal Slip	Prismatic Slip	Pyramidal Slip	Extension Twinning
Basal slip	0.04	0.04	0.04	0.02
Prismatic slip	1.6	1.6	1.6	0.02
Pyramidal slip	1.7	1.7	1.7	0.02
Extension twinning	0.02	0.02	0.02	0.6

## Data Availability

The data presented in this study are available on request from the corresponding author.
